# Automated identification of contextually relevant biomedical entities with grounded LLMs

**DOI:** 10.1038/s41598-026-35492-8

**Published:** 2026-01-13

**Authors:** Manuel Watter, Claudia Giuliani, Gita Benadi, Felix Engel, Harald Binder, Klaus Kaier

**Affiliations:** 1 Institute of Medical Biometry and Statistics, Medical Faculty and Medical Center, University of Freiburg, Freiburg, Germany; 2 Center for Integrative Biological Signaling Studies (CIBSS), University of Freiburg, Freiburg, Germany

**Keywords:** Medical research, Molecular medicine, Nephrology

## Abstract

This study investigates the effectiveness of different large language models for automated biomedical entity annotation in research articles with a focus on contextualized and grounded results. A 4-step generative workflow iteratively generates and refines entity candidates by considering a metadata schema for context and agentic tool use for validation with the PubTator 3 data base. The precision of this flow was assessed with a random effects meta-analysis after face-to-face interviews with authors of six papers from the Collaborative Research Center (CRC) 1453 “NephGen”. With an overall precision of 91.3%, the selected models provide qualitatively valuable annotations, with models GPT-4.1, GPT-4o Mini, and Gemini 2.0 Flash showing the highest precision. While GPT-4.1 and Gemini 2.0 Flash excelled in the total number of correct annotations, GPT-4o Mini and Gemini 2.0 Flash were fastest and most cost-effective. Large variations in annotation count and the conflation of publication and dataset-specific annotations highlight that human review ("human-in-the-loop") is still important. The results further highlight the trade-offs between precision, total number of correct annotations, cost, and speed. While quality is paramount in collaborative research settings, cost-effectiveness could be more critical in public implementations.

**Supplementary Information:**

The online version contains supplementary material available at 10.1038/s41598-026-35492-8.

## Introduction

Long-term research institutions such as Germany’s Collaborative Research Centers (CRCs) rely on structured data sharing and interoperable metadata to ensure long-term data reusability, in line with the FAIR principles^1^. In biomedical research, extracting entities such as organisms, cell lines, or genes from datasets and publications to create searchable tags improves discoverability and reuse. Because manual annotation is time-consuming, automated approaches such as named entity recognition are employed to identify and classify terms (e.g.. tagging “mouse” as an “organism”)^2^. Large Language Models outperform traditional approaches due to their ability to consider context in an unstructured text^3,4^.

Determining “how many” or even “which” entities should be extracted is ultimately an ill-posed question: relevance is shaped by the scientific context and the purpose of the downstream analysis. Moreover, entity relevance shifts with the experimental focus: cell-line names, for instance, are crucial when comparing in-vitro assays, but may be noise for population-level genomic studies. Consequently, optimal entity selection becomes a moving target that must be tuned to the downstream tasks. In our case, we have established a metadata schema in coordination with the experts of and for the CRC^5^. Until now, scientists had to manually annotate their data sets using this schema^6^. The schema will now serve as contextual guidance for the LLM as to „how many “ and „which “ entities to consider.

In recent years, there has been a shift from fine-tuning to in-context learning (ICL) as key strategies for adapting Large Language Models (LLMs) to downstream tasks. ICL guides the model using examples embedded directly in the input prompt, without modifying its internal parameters^7^. In contrast, fine-tuning updates the model’s parameters through additional training on task-specific data, which requires significantly more effort.

With the emergence of long-context LLMs, ICL has shown improved generalization over fine-tuning^7^. These extended context capabilities also enable integrations such as cache-augmented generation (CAG) and multi-step reasoning with tool use for complex tasks^8^. CAG involves preloading relevant data into the model’s context and caching parameters, allowing inference without further retrieval. This is especially effective when the knowledge base is compact and well-defined. We utilize the concept of preloading by providing the full text of a publication in the first message, which can save processing cost in our multi-turn workflows. As we compare multiple models through the OpenRouter API, the caching is ultimately in the hands of the respective providers.

The strength of modern LLMs as sophisticated, stochastic text generators entails their biggest downside: the “hallucination” of non-existent entities or factually incorrect statements. In a biomedical context, made-up proteins or gene-disease links are hard to spot, even for experts. Hence a mechanism is needed to reconcile predictions with reality, which is known as grounding^9^. Our approach exploits the increased capability of current models to reliably use external “tools”, by verifying LLM-suggested terms with the help of the PubTator 3 API^10,11^. Because it uses a controlled vocabulary, we also overcome the difficulties of synonyms or other notation-related errors.

A four-step methodology for annotation was established in Giuliani et al. 2025^10^, where the combined approach integrating in-context learning, providing said context and using external tools was first investigated. In this first study, predicted annotations achieved an average precision of 98% after verification by domain experts^10^. However, this approach is limited by its implementation with a custom GPT instance using ChatGPT-4o in a browser session^10^. In the present paper, we build upon these insights and assess the feasibility of the four-step workflow for a number of large language models. We implemented a fully automated framework based on API calls, with which we evaluated 14 different LLMs, of which eight models consistently supported reliable execution of the annotation workflow. These eight LLMs were subsequently employed to extract metadata suggestions for six research articles from CRC 1453 “NephGen”. The code for our framework and the data are available in the GitHub repository, https://github.com/watterm/llm-metadata-annotation.

## Methods

We base the methodology upon our experimental platform for few-shot metadata prediction. It implements a structured approach to information extraction from scientific publications with multi-turn LLM conversations.

**Fig. 1:**
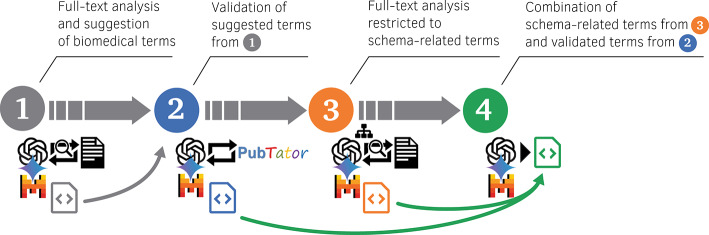
4-step generative workflow.

The four turns in a single conversation are as follows (Fig. 1). First, the model is instructed to extract relevant entities from the full text while disregarding the discussion and bibliography. Second, we direct the model to validate its suggested entities with the provided PubTator 3 tool. Third, we ask the LLM to consider and assign identified entities in context to a predetermined, hierarchical entity structure, using the metadata schema previously developed for CRC “NephGen”^5^. In the final turn, the model is ordered to consolidate the results of all previous steps. An example conversation is available online, https://watterm.github.io/llm-metadata-annotation/example. The structured JSON output in this example, particularly in steps 3 and 4, also serves as a practical visualization of the hierarchical metadata schema that guides the annotation process. In each turn we expect a JSON list as output, for which we require the model provider to support the Structured Output feature. This guarantees either a JSON output following our specifications or an error message. The sequential output of these lists with generated entity candidates allows the LLM to refine them based on its previous iteration in each step, but also to collate them repeatedly with the publication text. Hence, removing or adding entities can occur in each step while new context is introduced. This enables a “chain-of-thought” refinement towards contextually relevant and grounded entities.

Turn two requires the Tool Use feature from providers listed on OpenRouter. We define a “PubTator Search” tool, which queries the PubTator 3 API^11^, when invoked. Parsed and cleaned results are presented to the LLM. We enforce that the tool has to be used at least once. By storing these calls, we can evaluate if unqueried entity IDs were either hallucinated (not listed in PubTator 3) or inferred from the context with the model’s intrinsic knowledge (listed in PubTator 3). All experiments were conducted between April and May 2025. The specific model versions were those available through the OpenRouter API at that time. Our code, including the versions of all Python libraries used, is available in the linked GitHub repository to ensure maximum possible reproducibility.

Subject of our evaluation are six articles from the CRC “NephGen”, published between the first and the second funding period, each authored by a scientist who agreed to participate in a face-to-face interview. This criterion was intentionally chosen, as we believe that the presence of a research data management team member provides the necessary social control to ensure a reliable and diligent evaluation of the automated annotation. The articles were selected to represent a cross-section of the CRC’s research, including topics ranging from molecular mechanisms of kidney fibrosis to genetic studies of glomerular diseases, thereby covering different experimental models and techniques. During the interviews, all participants thoroughly reviewed the final LLM predictions, classifying them as correct or incorrect, while being allowed to refer to the article’s methods and results sections in case of uncertainty. Each review was limited to five minutes, to balance speed and reliability. Given the impossibility of having a ground truth, we can only calculate the precision of an annotation based on the expert’s judgment. Note that performance metrics such as recall and F1-score are not computable in this context, as the total number of potentially correct entities is not predefined and depends on the desired granularity of annotation.

To estimate precision across articles, we applied meta-analysis methods for single proportions^12^. Given the small number of entities per study, the Freeman-Tukey double arcsine transformation was used to stabilize variances, enhance robustness when proportions approach 1, and accommodate small sample sizes^13^. In other words, this transformation helps to properly analyze proportions (like our precision metric) by preventing statistical issues that can arise when values are very high (close to 100%) or when the number of data points is small, ensuring our analysis is more stable and reliable. A random-effects model with the REML estimator was used to account for between-study variance. Differences between LLMs were evaluated using random effects meta regression among transformed values. Differences in the costs, computation time and the total number of biomedical entities between LLMs were estimated with linear mixed models with a random intercept at the study level. All pairwise comparisons between LLMs were considered exploratory, and we report effect sizes with 95% confidence intervals to aid interpretation.

We initially selected 14 LLMs of various parameter counts and modalities (e.g. self-hostable), which met our feature criteria. Note that features such as Tool Use and Structured Output need to be implemented by providers, so availability depends not only on the models capabilities. After preliminary experiments, we removed five models, which were not able to reliably complete full conversations in our workflow. These included models that repeatedly failed to generate valid JSON or follow tool-use instructions (see Supplemental Fig. 1 for a detailed breakdown of model failures). For the interviews, we had to restrict the selection to five models (with six articles each) due to scientists’ time constraints. The three additional models were re-evaluated based on the existing classifications.

## Results

**Fig. 2:**
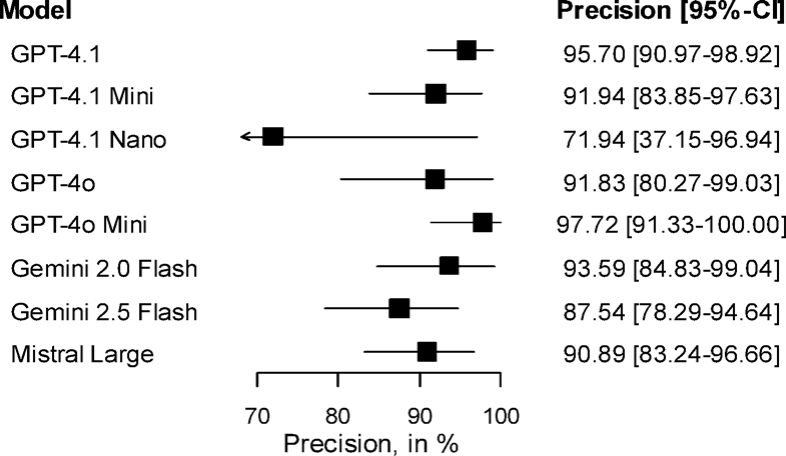
Precision of the different LLMs.

All LLMs performed reasonably well with an overall precision (defined as ratio of correctly identified entities over total number of annotation suggestions) of 91.3% [95%CI 87.4%-94.6%], meaning that the vast majority of predicted biomedical entities were considered correct in the face-to-face interviews. As shown in Fig. 2, GPT-4.1, GPT-4o Mini and Gemini 2.0 Flash were associated with the highest precision (95.7%, 97.7% and 93.6%), while GPT-4.1 Nano was associated with the lowest precision (71.9%). None of the differences between LLMs with the highest precision (GPT-4.1, GPT-4o Mini and Gemini 2.0 Flash), however, did reach statistical significance (p = 0.516 for the comparison of GPT-4o Mini and Gemini 2.0 Flash, for instance) and can therefore be in the range of randomness.

Estimates shown with 95% confidence intervals in brackets based on meta-analysis methods (precision) and linear regression models (time, costs and mean number of annotations).

**Fig. 3:**
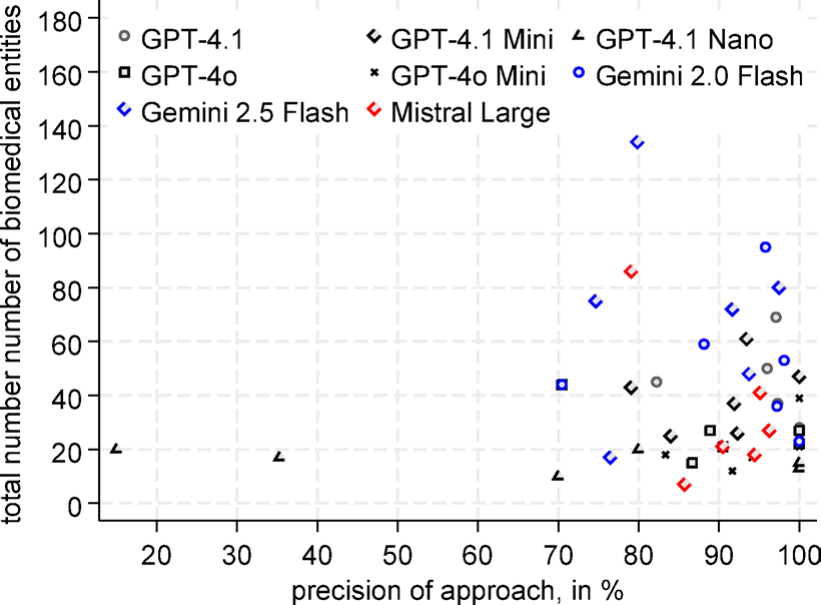
Precision of the different LLMs.

Overall, Gemini was the most diligent when it came to proposing biomedical entities. The mean number of correct entity annotations here was 47.5 and 60.8 for Gemini 2.0 Flash and Gemini 2.5 Flash, respectively. GPT-4o and GPT-4o Mini, in contrast, only proposed a mean of 22.8 and 21.0 entities per article (Table 1). As can be seen in Fig. 3, the total number of LLM-suggested biomedical entities was not correlated with the precision of the respective approach (p = 0.710). On the contrary, both GPT-4.1 and Gemini Flash 2.0 have a high rate of suggested entities with high precision (Table 1).

**Table 1:** Results of the 4-step approach.

	GPT-4.1	GPT-4.1 mini	GPT-4.1 nano	GPT-4o	GPT-4o mini	Gemini 2.0 flash	Gemini 2.5 flash	Mistral large
Time, in seconds	79.8	118	34	75.7	99.7	44	122.4	141.7
	[53.5,106.2]	[73.6,162.4]	[30.1,38.0]	[53.8,97.6]	[55.5,143.8]	[25.5,62.4]	[28.9,215.9]	[42.4,241.1]
Costs, in $	0.246	0.052	0.01	0.258	0.016	0.016	0.034	0.257
	[0.128,0.364]	[0.025,0.079]	[0.005,0.015]	[0.150,0.366]	[0.008,0.024]	[0.008,0.024]	[0.009,0.058]	[0.122,0.391]
Mean of correct annotations	44.0	36.2	10.0	22.7	21.0	47.3	60.8	29.2
	[29.7,58.3]	[21.8,50.5]	[4.4,15.6]	[16.1,29.3]	[10.6,31.4]	[21.8,72.9]	[27.6,94.1]	[6.2,52.2]
Precision, in %	95.70	91.94	71.94	91.83	97.72	93.59	87.54	90.89
	[90.97–98.92]	[83.85–97.63]	[37.15–96.94]	[80.27–99.03]	[91.33–100.0]	[84.83–99.04]	[78.29–94.64]	[83.24–96.66]

**Fig. 4:**
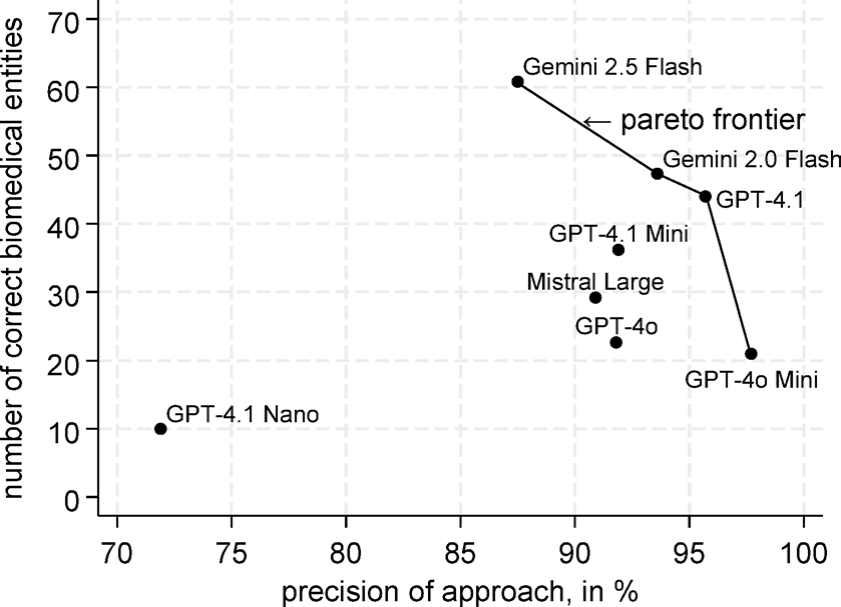
The Pareto frontier with respect to precision and number of correct predictions.

In Fig. 4, we see the Pareto frontier of our approaches with respect to precision and number of correct predictions. In this case, GPT-4.1, GPT-4o Mini, Gemini 2.0 Flash and Gemini 2.5 Flash are Pareto efficient compared to the other approaches.

Besides the scientific relevance measured by the interview, we evaluated the grounding with PubTator 3 in step 2 of the workflow. Supplemental Fig. 1 illustrates the efficacy of tool use for different model groups. As in the interview verification, the Gemini models predict a consistently high number of grounded entities with hallucinations occurring only for Gemini 2.5 Flash. Despite the naming scheme, performance did not increase from 2.0 to 2.5 or from Flash to Pro. Such improvements, however, can be seen for the GPT model class, where 4.1 and its Mini variant outperformed the older 4o models. The distillation to the Mini variant shows even a slightly better entity identification and the list shrunk significantly for GPT-4.1 Nano, which produced the fewest grounded entities among all tested models (see Supplemental Fig. 2 for a comparison of grounded, hallucinated, and inferred entities per model). Overall, the rate of false positives was low for models that could successfully complete a conversation.

**Fig. 5:**
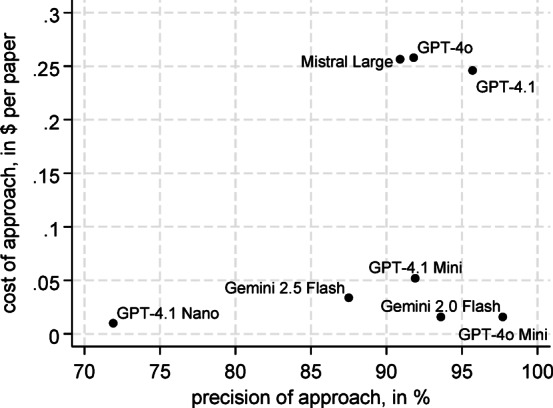
The precision and costs of LLMs.

There were considerable differences in the costs of the LLM execution (Fig. 5). Here, Gemini 2.0 Flash and GPT-4.1 Nano were associated with the lowest costs. At the time of the study, the costs here were $0.1 per million input tokens and $0.4 per million output tokens, respectively. On average, this resulted in costs of only $0.014 and $0.010 for the execution of the 4-step workflow with Gemini 2.0 Flash and GPT-4.1 Nano, respectively. GPT-4o Mini, Gemini 2.0 Flash and GPT-4.1 Mini, were nearly as favorable, with million token costs (input/output) of $0.15/$0.6, $0.15/$0.6 and $0.4/$1.6, respectively. GPT-4.1, GPT-4o and Mistral Large were in the upper range in terms of cost ($2/$8, $2.5/$10 and $2/$6, respectively) and all led to correspondingly higher costs per annotation. As shown in Fig. 5, GPT-4o Mini is the sole Pareto efficient model with respect to interview precision and cost. Interestingly, the cost differences between the two models with the highest precision (GPT-4.1 and GPT-4o Mini) was more than tenfold ($0.246 vs. $0.016, p < 0.001).

**Fig. 6:**
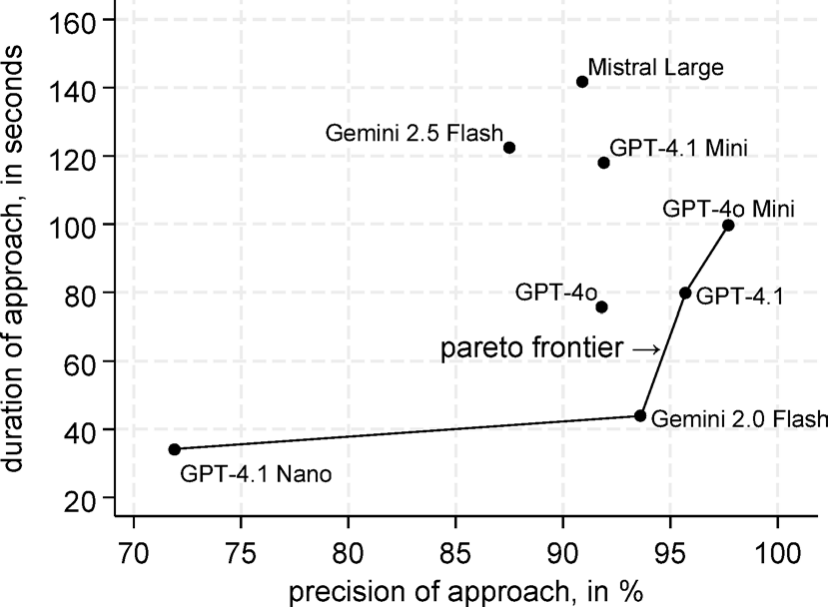
The Pareto frontier with respect to precision and duration of the LLM processing.

The same is true for the duration of the LLM processing. This was measured as the time between the request for every step and the corresponding response, excluding the tool call communication. GPT-4.1 Nano and Gemini 2.0 Flash were associated with the fastest execution time. As shown in Fig. 6, Mistral Large needed, on average, more than three times as long as Gemini 2.0 Flash. The Pareto frontier of our approaches with respect to precision and duration shows that GPT-4.1 Nano, Gemini 2.0 Flash, GPT-4.1 and GPT-4o Mini are Pareto efficient compared to the other approaches.

## Discussion

Overall, the investigated LLMs predicted biomedical entities quite reliably. Only GPT-4.1 Nano showed some downward outliers in its precision. We had previously observed similar results with Mistral Small and Mistral Medium, which, when run successfully, appeared less reliable than Mistral Large. In addition, both Mistral Small and Mistral Medium regularly failed to complete conversations, which is why they were not included in the interview evaluation. GPT-4.1 Nano performed the workflow reliably, but the results of at least one run suggest that the model is too small for the task at hand. Surprisingly, we found that the larger GPT-4.1 and GPT-4o ran faster than their “mini” counterparts, despite having a larger number of identified biomedical entities. In our opinion, this can only be explained by resource prioritization for the significantly more expensive models by their respective providers. A critical challenge for reproducibility is the reliance on external APIs, as providers can update models or underlying infrastructure without notice. This “black box” nature makes it difficult to guarantee identical results over time. Indeed, our experiments were occasionally delayed by bugs in provider SDKs related to tool use and structured output, highlighting the volatility of the development ecosystem. While the OpenRouter API is technically versioned, as indicated by “v1” in the URLs, their services were rapidly changing and undocumented updates required frequent changes during our development. Documenting the timeframe of experiments and software versions, as we have done, is currently the best practice for mitigating this challenge.

The multidimensional decision problem of LLM choice was taken into account by means of the Pareto frontiers. The precision was chosen as the primary decision criterion. The total number of correctly predicted biomedical entities can be seen as a secondary criterion. The large gap between GPT-4.1, Gemini 2.0 Flash and GPT-4o Mini shown here does not make the final decision easy. The speed and costs of the respective approach were considered tertiary in this study. Collaborative research centers focus on quality and processing time is of marginal relevance. In a public implementation of the presented approach, energy consumption^14–16^ and cost-effectiveness of the respective LLMs could become more important^17,18^. Here, self-hosting locally available open-source LLMs would be more cost-effective than the LLM-as-a-Service strategy we have chosen^19^. While quantifying the energy consumption of the models tested in this study was not feasible due to their API-based delivery, we consider the cost an important metric for future evaluations, especially when comparing with self-hosted alternatives. Unfortunately, at the time of our study, we could not identify any self-hostable open-source models that reliably supported the required tool use and structured output features, which precluded their inclusion in our automated workflow. As the open-source ecosystem continues to mature, we anticipate this limitation will diminish.

We may conclude that the applied LLM annotation works well and has the potential to accelerate the annotation of papers and datasets. A key challenge, however, is distinguishing between articles and their underlying datasets. This issue is a common challenge in automated information extraction from scientific literature and further underscores the current necessity of expert oversight to ensure the final annotations are directly relevant to the dataset itself. While metadata prediction is article-based, annotations should target datasets to facilitate their reuse. Beside possibly conflated information about the respective datasets, the publication also addresses the scientific context it contributed to, which entails the use of entities that are irrelevant to data annotation. To mitigate this discrepancy, we instructed the LLMs to disregard the discussion and bibliography sections during the prediction phase (step 1). Despite this measure, some incorrect predictions suggest that this part of the approach may not have been fully effective. Nonetheless, we consider it essential to apply and refine entities using the full text of each article, rather than limiting the input to selected sections. This strategy appears to be a more viable path towards establishing a fully automated annotation process, because it provides relevant information to the LLM instead of censoring the input.

As we have shown, the number of suggested biomedical entities varied substantially across LLMs, which makes the choice of a specific LLM elementary. For now, we highly recommend that LLM-assisted metadata annotations should undergo human review before publication ("human-in-the-loop"). This could be both technically enforced and systematically integrated into the workflow.

A major limitation is the small sample size of six articles from a single Collaborative Research Center. This focused approach was chosen to ensure high-fidelity validation through direct author interviews but restricts the generalizability of our findings. While the selected papers covered a range of topics within nephrology, the robustness of the workflow and the relative performance of the LLMs need to be confirmed on a larger and more diverse corpus of biomedical articles from different fields. Future work should employ more scalable validation methods to assess performance across a wider variety of publication types and scientific domains. A further limitation of the approach is the inability to identify biomedical entities that were not predicted by the LLMs. In other studies on natural language processing with LLMs, false negatives are commonly taken into account when calculating performance metrics such as accuracy, recall, and the F1 score. However, in the present context, where the primary objective is to maximize the number of correct annotations while minimizing the proportion of false positives, this approach is not feasible^10^. The key limitation lies in the fact that the number of potential annotations per dataset does not have a clearly defined upper bound. While it would, in principle, be possible to ask authors during face-to-face interviews which biomedical entities they believe are missing, obtaining this information would require considerable additional effort. Nevertheless, since our approach deliberately guides the LLM by providing it with the predefined CRC metadata schema, it at least ensures that the generated annotations are not only contextually appropriate for the given paper but, more importantly, are also relevant in relation to the CRC framework.

To address the methodological limitations of this study, future research could attempt to approximate recall by establishing a consensus ground truth. This reference standard could be built longitudinally by capturing expert feedback during a routine implementation of the annotation tool. Within such a workflow, the human-in-the-loop process would involve experts not only correcting or removing false positives but also adding any false negative (entities the model failed to identify). Such a benchmark, aggregated over many interactions, would make it possible to approximate recall and calculate F1-scores, thereby providing a clearer picture of the models’ ability to identify a comprehensive set of entities. Furthermore, this ground truth would allow for a quantitative analysis of the "human-in-the-loop" component. By comparing the raw LLM outputs against this reference set, we could measure the models’ standalone performance, while a subsequent comparison with the final, expert-curated annotations would precisely quantify the improvement gained through human review. This analysis would clarify the necessity and impact of human oversight and help in designing more efficient and targeted review processes.

## Supplementary Information

Below is the link to the electronic supplementary material.

Supplementary Material 1

Supplementary Material 2

## Data Availability

The software is available in the GitHub repository: https://github.com/watterm/llm-metadata-annotation The datasets used and/or analysed during the current study are available from the corresponding author on reasonable request.
