# Supplementary figures and images for: Automated identification of contextually relevant biomedical entities with grounded LLMs
Source: Sci Rep. 2026 Jan 13;16:1952. doi: 10.1038/s41598-026-35492-8 (PMC12804813; doi:10.1038/s41598-026-35492-8)

Supplemental Figure 2: Results of the meta-analyses

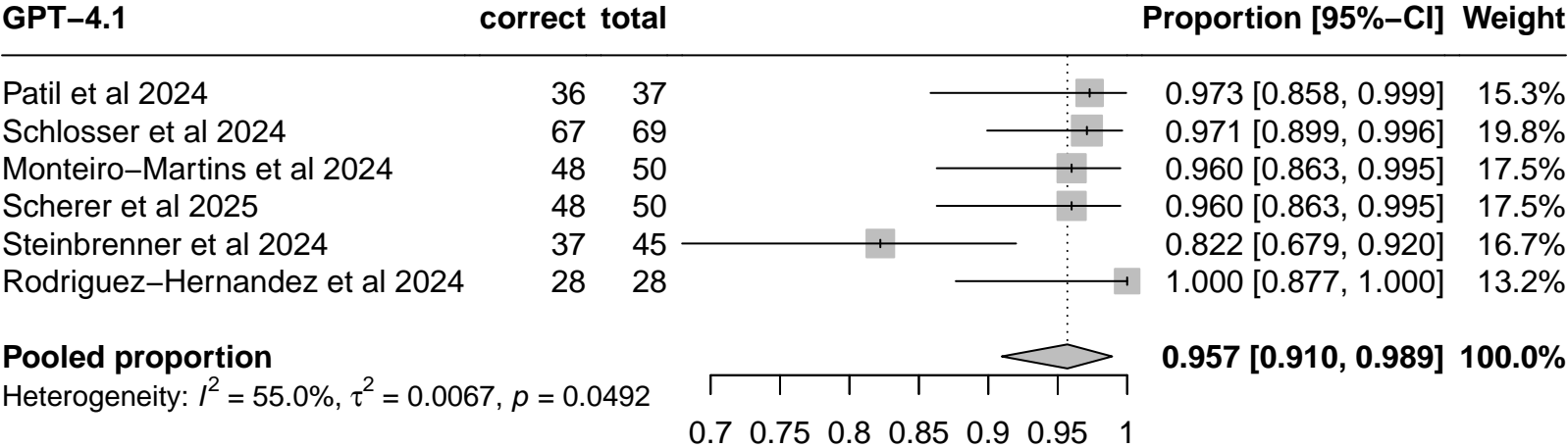

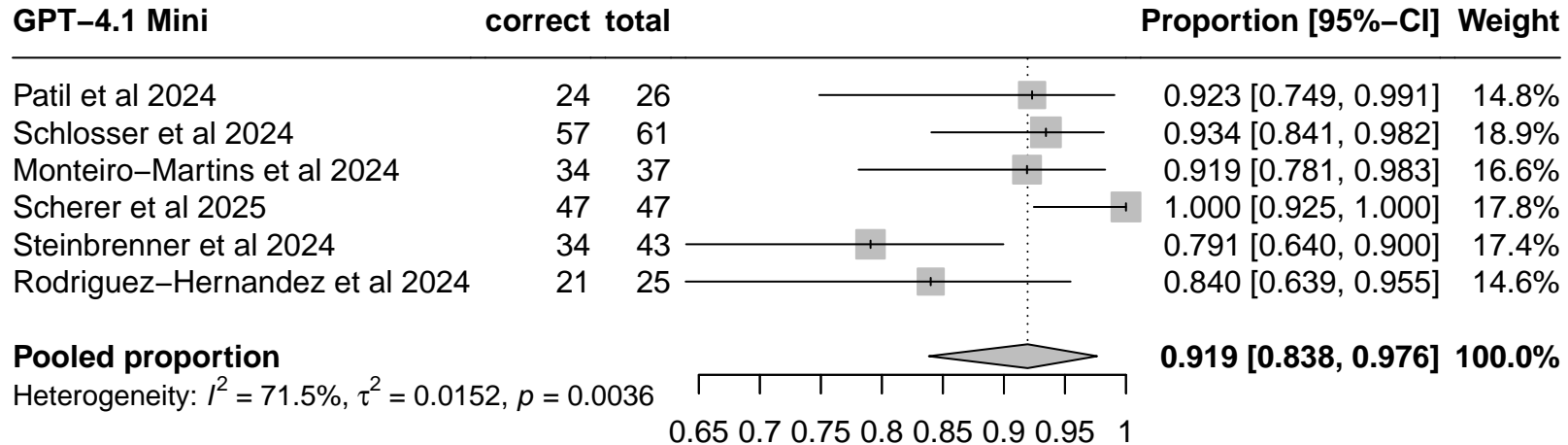

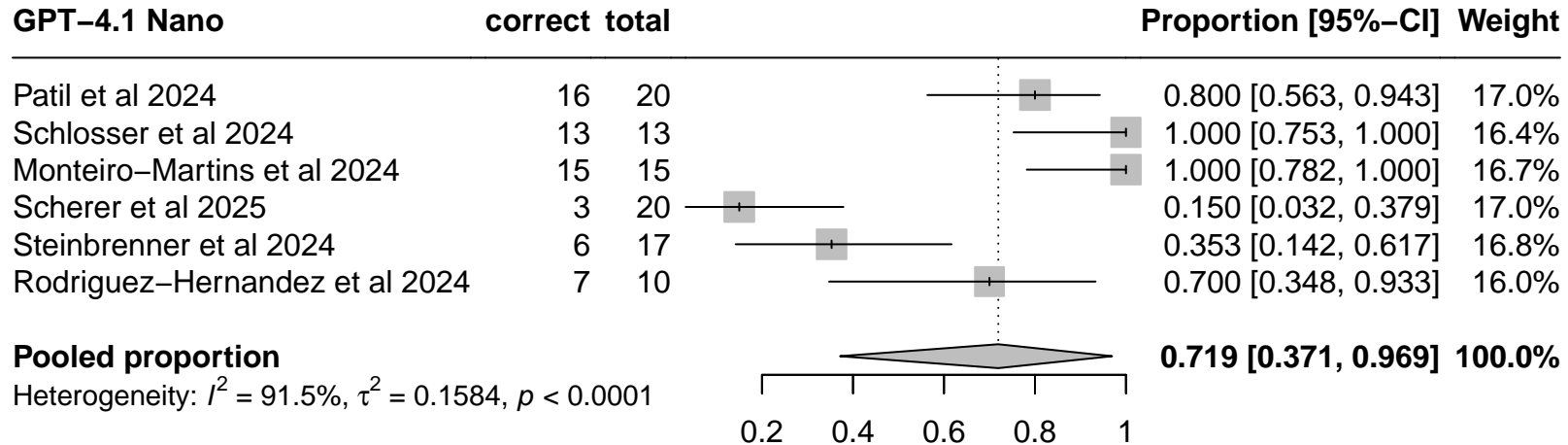

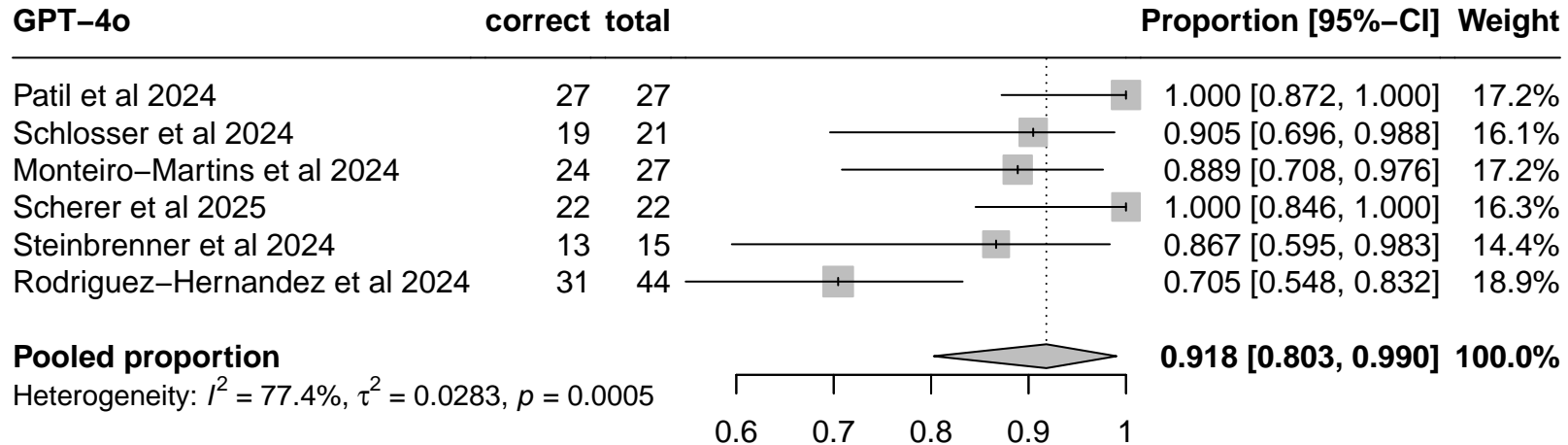

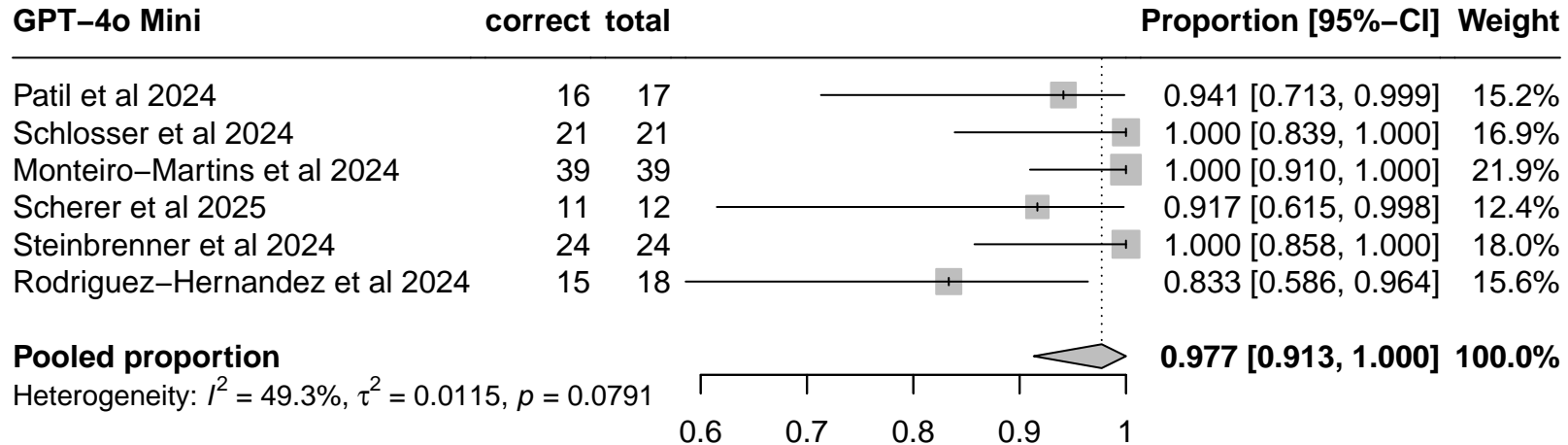

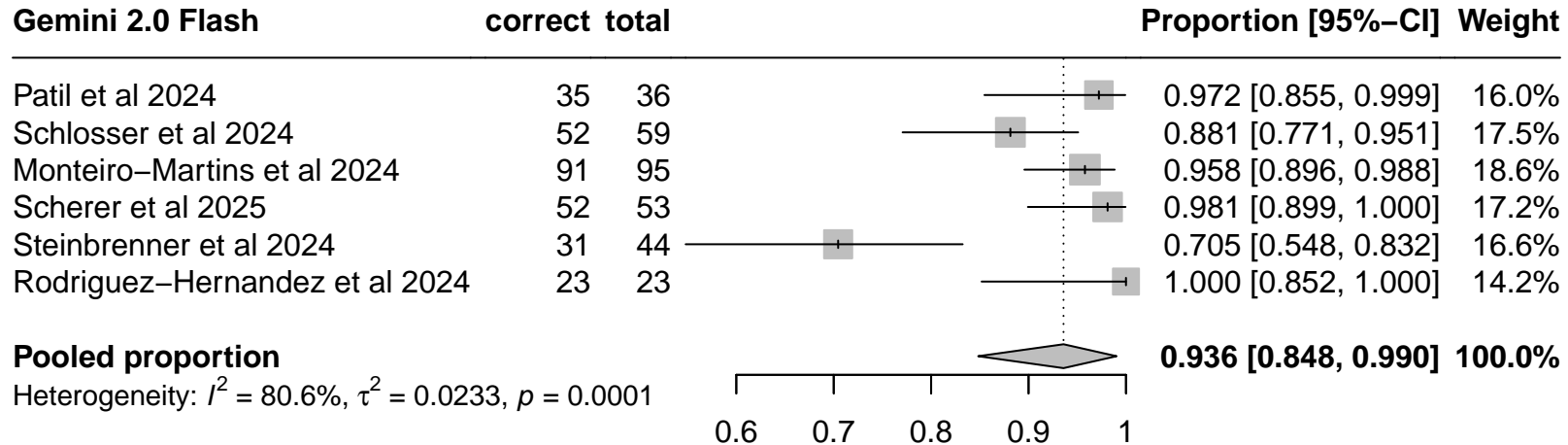

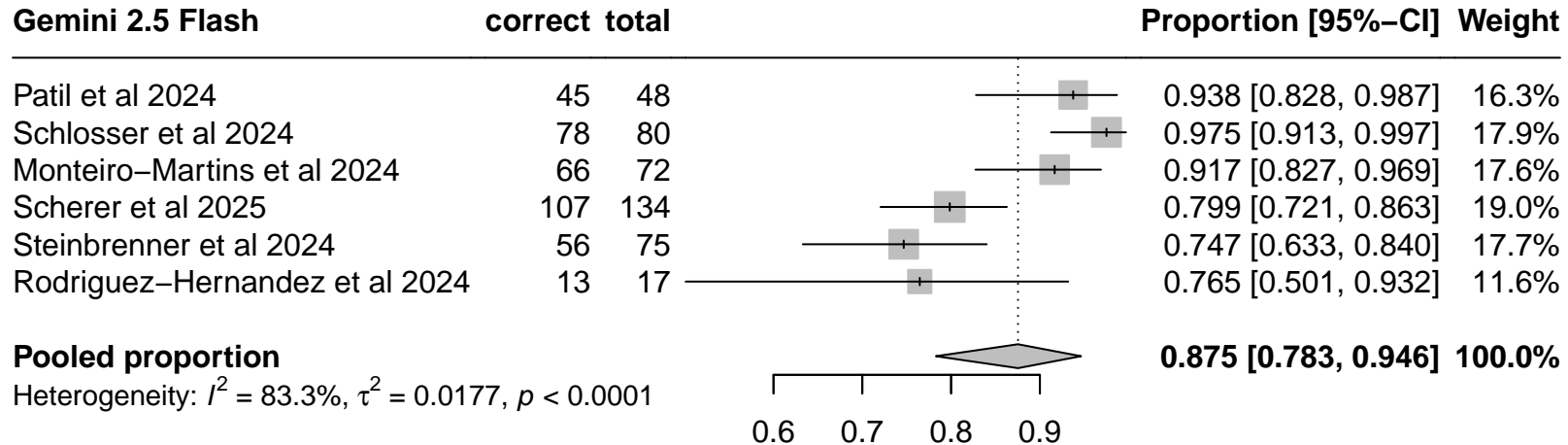

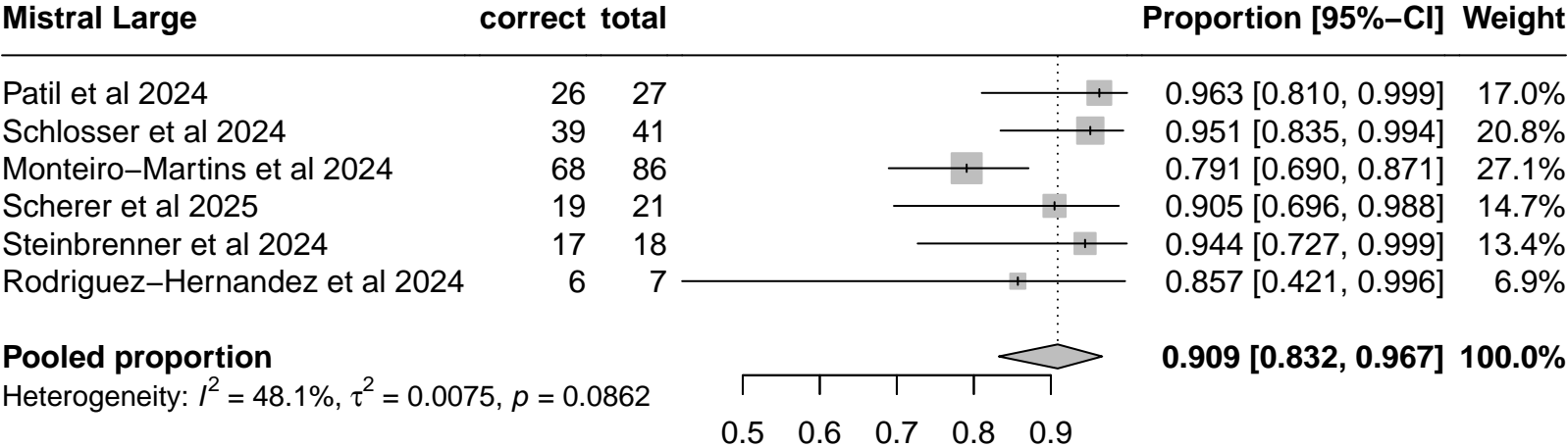

Supplement: Supplementary file 1 — Supplementary Material 1 [file 41598_2026_35492_MOESM1_ESM.pdf]
