# Supplementary material for: Automated identification of contextually relevant biomedical entities with grounded LLMs
Source: Sci Rep. 2026 Jan 13;16:1952. doi: 10.1038/s41598-026-35492-8 (PMC12804813; doi:10.1038/s41598-026-35492-8)
Supplement: Supplementary file 2 — Supplementary Material 2 [file 41598_2026_35492_MOESM2_ESM.pdf]

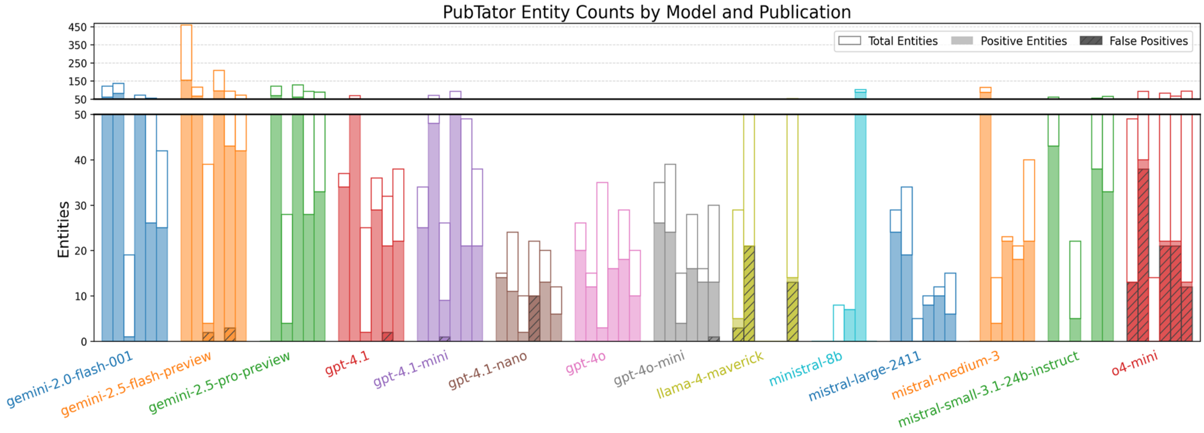

Supplemental Figure 1: The cardinality of the entity set after step 2 for each model and each publication. Each bar indicates how many entities were listed in total (step 1), positively predicted to be in PubTator 3 with an ID (step 2) and how many were hallucinations and do not exist in the database. Missing bars indicate failed. Finally, we were able to build a stable pipeline for operationalizing the described four-step approach with eight LLMs: Google Gemini 2.0 Flash 001, Gemini 2.5 Flash (2025-04-17), OpenAI GPT 4o (2025-03-26), OpenAI GPT 4o mini (2024-07-18), Mistral Large 2411, OpenAI GPT 4.1 mini (2025-04-14), OpenAI GPT 4.1 nano (2025-04-14) and OpenAI GPT 4.1 (2025-04-14).
